# UWB-Modulated Microwave Imaging for Human Brain Functional Monitoring

**DOI:** 10.3390/s23094374

**Published:** 2023-04-28

**Authors:** Youness Akazzim, Marc Jofre, Otman El Mrabet, Jordi Romeu, Luis Jofre-Roca

**Affiliations:** 1 School of Telecommunication Engineering, Universitat Politècnica de Catalunya, 08034 Barcelona, Spain; marc.jofre@upc.edu (M.J.); jordi.romeu-robert@upc.edu (J.R.); luis.jofre@upc.edu (L.J.-R.); 2 System of Information and Telecommunications Laboratory (LaSIT), FS, Abdelmalek Essaadi University, Tetouan 93000, Morocco; oelmrabet@uae.ac.ma; 3 Department of Research and Innovation, Hospital General de Granollers, 08402 Granollers, Spain

**Keywords:** UWB microwave imaging, action potential, microtag, UWB microwave modulation, functional imaging, Parkinson’s disease

## Abstract

Morphological microwave imaging has shown interesting results on reconstructing biological objects inside the human body, and these parameters represent their actual biological condition, but not their biological activity. In this paper, we propose a novel microwave technique to locate the low-frequency (f≃1 kHz) -modulated signals produced by a microtag mimicking an action potential and proved it in a cylindrical phantom of the brain region. A set of two combined UWB microwave applicators, operating in the 0.5 to 2.5 GHz frequency band and producing a nsec interrogation pulse, is able to focus its radiated field into a small region of the brain containing the microtag with a modulated photodiode. The illuminating UWB microwave field was first modulated by the low-frequency (f≃1 kHz) electrical signal produced by the photodiode, inducing modulated microwave currents into the microtag that reradiating back towards the focusing applicators. At the receiving end, the low-frequency (f≃1 kHz) -modulated signal was first extracted from the full set of the backscattered signals, then focused into the region of interest and spatially represented in the corresponding region of the brain, resulting in a spatial resolution of the images in the order of 10 mm.

## 1. Introduction

Microwave imaging is a competitive technique that is able to reconstruct biological parts inside a living body thanks to its penetration, compactness, safety, and cost advantages. Based on the reconstruction of the parameters such as the spatial profile of the permittivity, it is able to differentiate different tissue compositions [1,2], morphologies [3,4,5,6,7], and eventually their physiological state (as for the case of cancer) [8,9,10,11]. These parameters represent their actual biological condition, but not their biological activity. When interested in looking at the functional (dynamic) activity, one of the potential ways is through the observation of the (low-frequency (f≃1 kHz)) electrical signals responsible for the different biological phenomena, normally identified as action or membrane potential signals (impulse responses with amplitudes normally in the order of hundreds of mVolts and frequencies in the order of kHz (msec durations)) [12,13]. Due to the low-frequency (f≃1 kHz) character of these signals, they stay mostly confined inside living bodies, and consequently, different kinds of contact electrodes have been normally used to visualize them [14,15]. Due to its informational content, there would be a major interest in extracting these low-frequency (f≃1 kHz) signals in a wireless and contactless way.

The functional information within the human body is widely used for healthcare applications, as cardiac action potentials to monitor the electrocardiograms [16,17,18,19,20]. Furthermore, the brain action potentials have been studied for rats [21] or humans [22] using the microwave transmission method to detect the neural activity. This method studies the variation of the propagating wave phase due to the changes in the values of the permittivity through the functional sites.

In this paper, we propose to explore the possibility of using a novel methodology to wirelessly monitor these low-frequency (f≃1 kHz) signals from the inside of the body using UWB signals. In this sense, a preliminary exploratory work in [23,24] was presented based on the capability of extracting these low-frequency (f≃1 kHz) biological signals as the modulating effect on an interrogating focused incident high-frequency microwave signal (carrier signal). The microwave signal is able to propagate inside (in and out) of the human body, being focused (with a UWB multi-probe geometry [25]) on a specific region of it, and eventually becoming affected (modulated) by the low-frequency (f≃1 kHz) functional biological signals. After obtaining the information from the action potential signal representing the functional activity, it is located back. In particular, the capability of monitoring localized action potential signals in the brain may help into the understanding of different functional anomalies as Parkinson’s disease (PKD), characterized by electrical signals generated in the subthalamic nucleus (STN) and the globus pallidus (GPi) [26] with specific action potential patterns.

The remainder of this paper is organized as follows. Section 2 provides the analytical basis of the UWB modulation technique. The initial numerical and experimental validation are then presented and discussed in Section 3, where the action potential is modeled by a photodiode located inside a phantom of the brain model to generate an electrical response that emulates the action potential. Finally, some preliminary conclusions are presented in Section 4.

## 2. Analytical Formulation

### 2.1. Illuminating–Focusing Geometry

**Figure 1:**
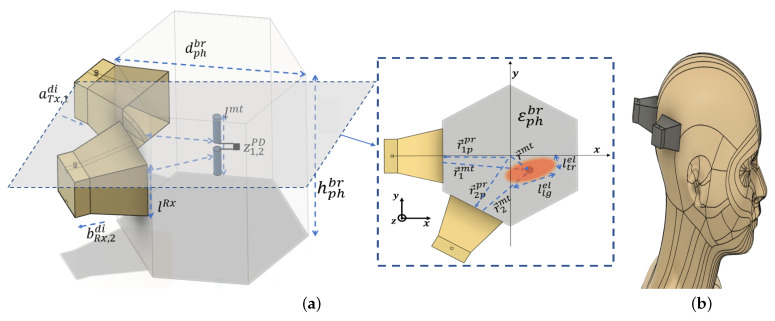
The principal of functional monitoring. (**a**) Modeling of the human brain. (**b**) General scenario for the functional microwave imaging.

The general geometry (Figure 1a) consists of a cylindrical phantom, modeling a human head (Figure 1b), filled with a dielectric medium representative of the brain’s average permittivity value. The model contains a miniature low-frequency (f≃1 kHz) -modulated diode emulating the signaling capability of a biological phenomenon and an UWB microwave illuminating antenna set with two EGRH horns (unidirectional UWB frequency operation) located at position ${\vec{r}}_{ip}^{pr}$ able to focus the interrogating microwave illumination into a reduced region (with dimensions in the order of the effective wavelength into the biological medium) inside the brain. A good compromise between complexity and resolution is obtained for a two-horn geometry [27].

In this scenario, two sequentially transmitting and receiving UWB horn antennas [28] covering the microwave $f_{lw}^{rf}$ to $f_{up}^{rf}$ frequency range create an illuminating focused beam spot over an ellipsoidal region with approximate transversal and longitudinal dimensions $l_{tr}^{el}$ and $l_{ln}^{el}$, respectively, containing a microtag (optically modulated photodiode with impedance state $Z_{ON}^{PD}$ and $Z_{OFF}^{PD}$) able to produce an equivalent action potential low-frequency (f≃1 kHz) $f_{ap}^{lf}$ switching signal located at the position ${\vec{r}}^{mt}$ with distances of $r_{1}^{mt}$ and $r_{2}^{mt}$ from the two probes inside the brain geometry with complex permittivity $\varepsilon_{ph}^{br}$.

### 2.2. RF UWB Scattering

In order to obtain the simultaneous spatial focusing and low-frequency (f≃1 kHz) signal extraction for the case of two Tx–Rx horns, a 2 × 2 matrix of S parameters was obtained as in [29]. According to the general scenario of Figure 1a, each of the two Tx probe antennas sequentially illuminate the full region under study, producing scattered returns from the different objects that will be collected by each one of the two Rx antennas. From these 2 × 2 set of signals, only the modulated term $\Delta S_{ij}^{md}\left(f\right)$ backscattered by the microtag antenna is retained, resulting from the two different impedances of the load as [30]

(1) $$\Delta S_{ij}^{md}\left(f\right)=S_{ij}^{L1}\left(f\right)-S_{ij}^{L2}\left(f\right)=G^{hrn}\left(f\right).G^{mt}\left(f\right).\frac{1}{r_{i}^{mt}}.\frac{1}{r_{j}^{mt}}.{\left(\frac{V_{br}^{ph}}{4.\pi .f}\right)}^{2}\left|{\tilde{\rho }}_{L_{1}}\left(f\right)-{\tilde{\rho }}_{L_{2}}\left(f\right)\right|$$

where $G^{hrn}$ and $G^{mt}$ are the gain of the horn antennas and of the microtag equivalent antenna, respectively, $V_{br}^{ph}$ is the wave velocity in the medium, and ${\tilde{\rho }}_{L_{i}}\left(f\right)$ is the complex reflection coefficient for the two states of the photodiode $Z_{i}^{PD}\left(f\right)$:

(2) $${\tilde{\rho }}_{L_{i}}\left(f\right)=\frac{Z_{i}^{PD}\left(f\right)-{\left(Z_{a}^{SD}\left(f\right)\right)}^{*}}{Z_{i}^{PD}\left(f\right)+Z_{a}^{SD}\left(f\right)}$$

where $Z^{SD}$ is the impedance of the connecting leads of the photodiode modeled as a short dipole and ${\left(Z_{a}^{SD}\left(f\right)\right)}^{*}$ denotes its complex conjugate.

The combination of the microwave UWB interrogation signal with the modulating low-frequency (f≃1 kHz) signal controlling the switching photodiode, may be then expressed in the time domain, extending the one-way expression in [31] to the two-way-modulated radar case, resulting in:

(3) $$\begin{array}{c} \Delta s_{ij}^{md}\left(t\right)=\frac{\Delta b_{Rx,j}^{di}}{a_{Tx,i}^{di}}=\frac{\Delta v_{Rx,j}^{-}}{v_{Tx,i}^{+}}=\left\{\frac{1}{4\pi v_{br}^{ph}r_{i}^{mt}}.l_{ef}^{pr}\left(\tau ,{\hat{r}}_{i}^{mt}\right)\right\}*m_{lf}^{mt}\left(t\right)*\\ \left\{l_{ef}^{mt}\left(\tau ,{\hat{r}}_{i}^{mt}\right)*\frac{\sqrt{\eta_{br}R_{a}}}{\left[Z_{a}^{SD}+Z_{L}^{PD}\left(t\right)\right]}\right\}*\left\{\frac{1}{4\pi v_{br}^{ph}r_{j}^{mt}}.l_{ef}^{pr}\left(\tau ,{\hat{r}}_{j}^{mt}\right)\right\}\\ {}*v_{Tx}^{{}^{\prime \prime }}\left(\tau -\frac{r_{i}^{mt}+r_{j}^{mt}}{v_{br}^{ph}}\right) \end{array}$$

where *i* and *j* are the transmitting and receiving probes respectively, $\eta_{br}$ the impedance of the brain medium, $a_{Tx,i}^{di}$, $b_{Rx,j}^{di}$, and $v_{i}\left(t\right)$ and $v_{i}^{\prime }\left(t\right)=\frac{dv_{i}}{dt}$, respectively, the voltages applied and radiated by the *i*-th transmitting antenna, $P_{T}$ the transmitted power for each of the two probes, and $m_{lf}^{mt}\left(t\right)$ the low-frequency (f≃1 kHz) time-modulating signal produced by the microtag device (the combination of the short dipole and switching photodiode). The unit vector directions from antenna Tx or Rx to the microtag are denoted, respectively, by ${\hat{r}}_{i}^{mt}$. It was assumed that the different effective lengths and the gain of the microtag (short dipole antenna) may be considered mostly constant in the frequency range of interest.

In order to produce the simultaneous UWB location and low-frequency (f≃1 kHz) detection processes, three sequential operations are applied: (i) the target-modulated signals are selected from the different scattered signals produced by the head environment; (ii) UWB bifocusing technique into the frequency domain or equivalently a time-reversal focusing algorithm in the time domain is applied, scanning the different points inside the reconstruction volume, taking advantage of the short pulse location capability; (iii) the remaining signal is then demodulated (passed through a low-pass filter matched to the low-frequency (f≃1 kHz) microtag modulation form approaching the biological impulse response).

### 2.3. UWB Inverse Mapping

In the previous section, Equation (3) calculates the time domain radiated scattering parameters $\Delta s_{ij}^{md}\left(t\right)$ for the two probes, where each element acts sequentially as the Tx or Rx. The equation expresses the voltages corresponding to the retarded fields produced by the scattering object (microtag) located at a certain specific location, producing a certain modulated signal. While scattering may come from different parts of the volume inside the explored region, the system will only retain those reflections being modulated with the low-frequency (f≃1 kHz) signal corresponding to the investigated process (i.e., Parkinson’s).

For the inverse imaging process, based on the UWB-bifocusing technique (UWB-BF) [32] or time reversal [33], we may express the focused image for every point of the mapping region ${\vec{r}}_{foc}$ in the frequency domain and in the time domain, respectively, as

(4) $$\begin{array}{c} I_{freq}^{sign}\left({\vec{r}}_{foc}\right)=A_{freq}\Sigma_{f_{lw}^{rf}}^{f_{up}^{rf}}\Sigma_{j=1}^{N_{R}}\Sigma_{i=1}^{N_{T}}\frac{1}{f^{2}}S_{ij}^{md}\left({\vec{r}}_{ip}^{pr},{\vec{r}}_{jp}^{pr},f\right)\frac{1}{G(\left[{\vec{r}}_{ip}^{pr}-{\vec{r}}_{foc}\right];f)}\\ \frac{1}{G(\left[{\vec{r}}_{jp}^{pr}-{\vec{r}}_{foc}\right];f)} \end{array}$$

The Fourier transform allows us to translate the frequency domain, resulting in

(5) $$\begin{array}{c} I_{time}^{sign}\left({\vec{r}}_{foc}\right)=A_{time}\Sigma_{t_{min}}^{t_{max}}\Sigma_{j=1}^{N_{R}}\Sigma_{i=1}^{N_{T}}s_{ij}^{md}\left({\vec{r}}_{ip}^{pr},{\vec{r}}_{jp}^{pr};t+\frac{\left[{\vec{r}}_{ip}^{pr}-{\vec{r}}_{foc}\right]\left[{\vec{r}}_{jp}^{pr}-{\vec{r}}_{foc}\right]}{v_{br}^{ph}}\right)\\ \frac{1}{\left[G\left(\left[{\vec{r}}_{ip}^{pr}-{\vec{r}}_{foc}\right];f\right)\right]}\frac{1}{\left[G\left(\left[{\vec{r}}_{jp}^{pr}-{\vec{r}}_{foc}\right];f\right)\right]} \end{array}$$

where $A_{freq}$ and $A_{time}$ are, respectively, the frequency and time domain complex constant, including multiple system factors. $N_{R}$ and $N_{T}$ are the number of transmitters and receivers.

**Figure 2:**
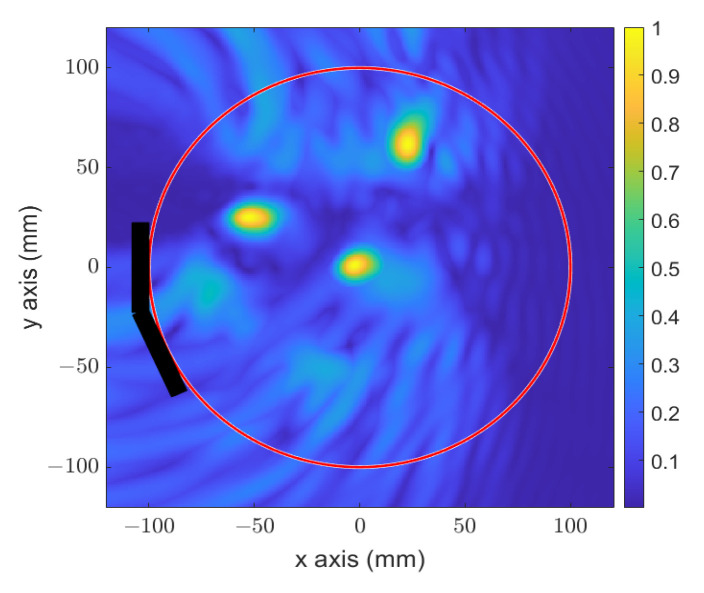
Numerical reconstructed image of three circular targets in the approached size of the human head.

In order to see the mapping capability of the proposed focusing technique, Figure 2 presents a numerical image of a circular geometry of 200 mm in diameter approaching the dimensions of the head, in which three small scattering points have been reconstructed, showing the focusing dimension in the order of less than a 10 mm diameter.

### 2.4. Scattering Low-Frequency (f≃1 kHz) Action Potential Modulation Process

In this study, the non-linear effect of the action membrane of the microorganism (dopaminergic neuron for the case of Parkinson’s application) was approached with the non-linear character of the photodiode used as a microtag.

To avoid any perturbation produced by the arrival of the modulating signal in the photodiode, a modulated ($f_{ap}^{lf}=1$ kHz) LED (TSUS4300 950 nm GaAs infrared-emitting diode) illuminates a 50 mm distant photodiode (WL-SDCB SMT with overall dimensions a 3.2 mm length, 1.5 mm width, and 1.2 mm height and equivalent active zone of 200μm×200μm). Overall, the modulated photodiode is then representative of the functional activity based on its non-linear effect and active zone size comparable to those of the membrane of the microorganism.

The encapsulated photodiode (microtag) may be modeled as a short dipole (package and leads) responsible for receiving and transmitting the RF microwave signals, and the active zone is responsible for the reception of the optical signal from the LED generating the low-frequency (f≃1 kHz) information of a duration 1 msec and a 200 mVolt impulse response modulating the microwave signal by changing the two states of the photodiode equivalent real part impedance $Re[Z_{ON}^{PD}]=3\;\Omega$ and $Re[Z_{OFF}^{PD}]=20\;k\Omega$ frequently with a $f_{ap}^{lf}=1$ kHz signal, to model the brain activity.

## 3. Numerical and Experimental Validation

**Figure 3:**
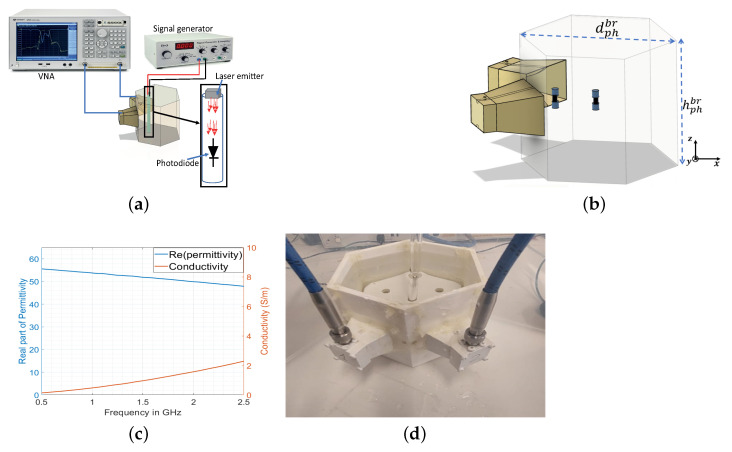
Functional monitoring setup. (**a**) Schematic for measurement process. (**b**) Schematic scenario of the simulation. (**c**) The measured permittivity of the fabricated liquid gel. (**d**) Measurement setup with liquid phantom.

In this section, the proposed approach was first numerically validated and then experimentally proven with a basic head–brain model. The idea was to spatially locate within the brain region the origin of the switching signal (produced by the photodiode; Figure 3a) mimicking a biological functional activity. The switching character of the functional signal was used to mark and differentiate the backscattered modulated signal from the rest of the non-modulated scattering returns. In Figure 3b is represented the system consisting of two EGRH UWB horn antennas [28] (filled with a material permittivity ($\varepsilon_{r}≃57$) mimicking the human brain medium to achieve a proper matching between the probes and the model), located in contiguous faces of a cylindrical hexagonal phantom with an internal diagonal length of $d_{ph}^{br}=100$ mm. The probes were connected to an E5071C vector network analyzer (VNA) to measure the signals $S_{ij}^{md}$ backscattered from the target, acting successfully as the Tx and Rx antennas and operating in the frequency range of $f_{lw}^{hf}=0.5$ GHz to $f_{up}^{hf}=2.5$ GHz, where the lower frequency limit was selected to achieve a good penetration into the human body and the higher frequency limit was selected based on the penetration range inside the human model [34]. The phantom was filled with a liquid gel approaching the brain average permittivity $\varepsilon_{r}^{{}^{\prime }}=57$, σ=0.6 S/m at 0.8 GHz [35]. The gel was fabricated using the combination of 50% distilled water and 50% methyl alcohol $99.9^{\circ }$ [27]. The resulting permittivity was measured using the N1501A dielectric probe kit and is presented in Figure 3c. Inside the phantom, a photodiode of length $l^{mt}=3.2$ mm (microtag) was located in two different positions along the bisector between the two horns (at distances of 0 mm and 30 mm from the center), as in Figure 3b. The experimental setup is presented in Figure 3d.

**Figure 4:**
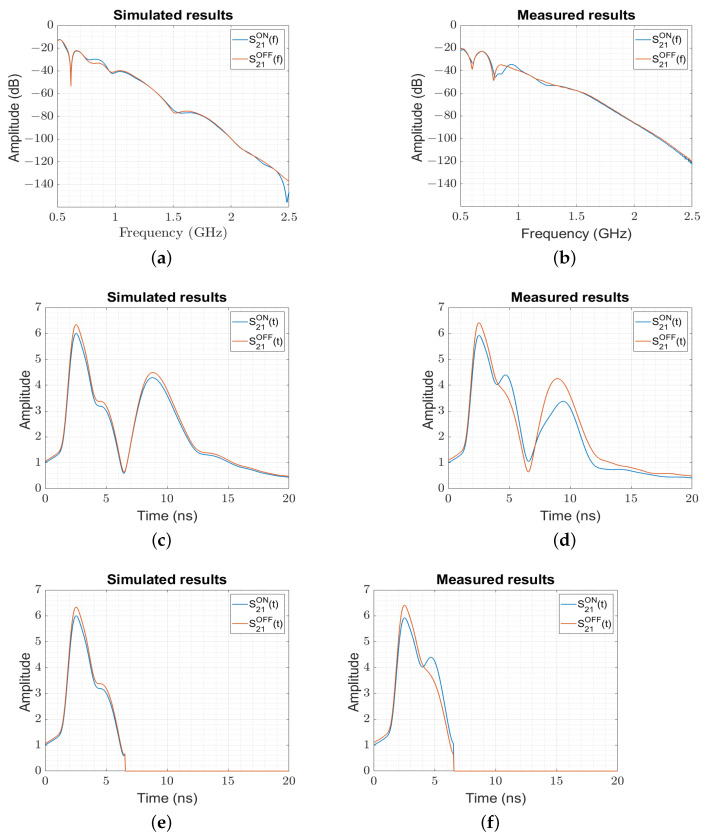
The simulated backscattered S21 parameter from the photodiode “ON” and “OFF” states. (**a**) The amplitude of the simulated S21 in the frequency domain. (**b**) The amplitude of the measured S21 in the frequency domain. (**c**) The amplitude of the simulated S21 in the time domain. (**d**) The amplitude of the measured S21 in the time domain. (**e**) The amplitude of the simulated S21 in the time domain after time filtering. (**f**) The amplitude of the measured S21 in the time domain after time filtering.

In Figure 4, we present the simulated and measured received backscattered signals from the microtag in the frequency (Figure 4a,b) and time domains (Figure 4c,d) for the two states of the photodiode.

Figure 4a,b present the $S_{21}$ parameters for the two states (ON/OFF) of the microtag that approach the small variation corresponding to the two states of the membrane potential.

In order to study the capability of the system to monitor (detect) a microorganism’s functional activity, we studied the modulated backscattered $S_{21}$ signals corresponding to the two states (ON and OFF) of the microtag and compared this measured level to the one of a representative microorganism. Therefore, we calculated the reflection coefficient using (2) for the actual microtag used in our experiment with the one of the microorganism. In Table 1, we present the experimental values of the backscattered modulation index (BMI) ($BMI=\frac{{\tilde{\rho }}_{L_{ON}}\left(f\right)}{{\tilde{\rho }}_{L_{OFF}}\left(f\right)}$) for the measured microtag (BMI≃0.040) (based on the ON and OFF levels extracted from Figure 4d) compared to the analytical values (BMI≃0.045), showing a very good agreement. When comparing these values to the ones corresponding to a representative microorganism, we may observe that, due to the smaller size of the microorganism (with an approximate surface of 200μm×200μm) responsible for the creation of the membrane potential, the corresponding BMI is around 10-times smaller (BMI≃0.005), corresponding to an 18 dB-lower BMI.

Inspecting the time domain representation of the backscattered signal for the simulated (Figure 4c) and measured (Figure 4d) setup (obtained by using (3), which contains the modulating signal produced by the microtag device $m_{lf}^{mt}\left(t\right))$, two peaks may be observed, where the first at $t_{p}^{mt}≃2.5$ ns corresponds to a distance of $r_{p}^{mt}=50$ mm from the open end of the horn and the second at around $t_{p}^{mt}≃9$ ns corresponds to the reflection from the hexagonal wall. The time filtering technique (time information above the limits of the walls was discarded, and the values were put to zero) was applied to remove the undesired signals that were above the wall (Figure 4e,f).

**Figure 5:**
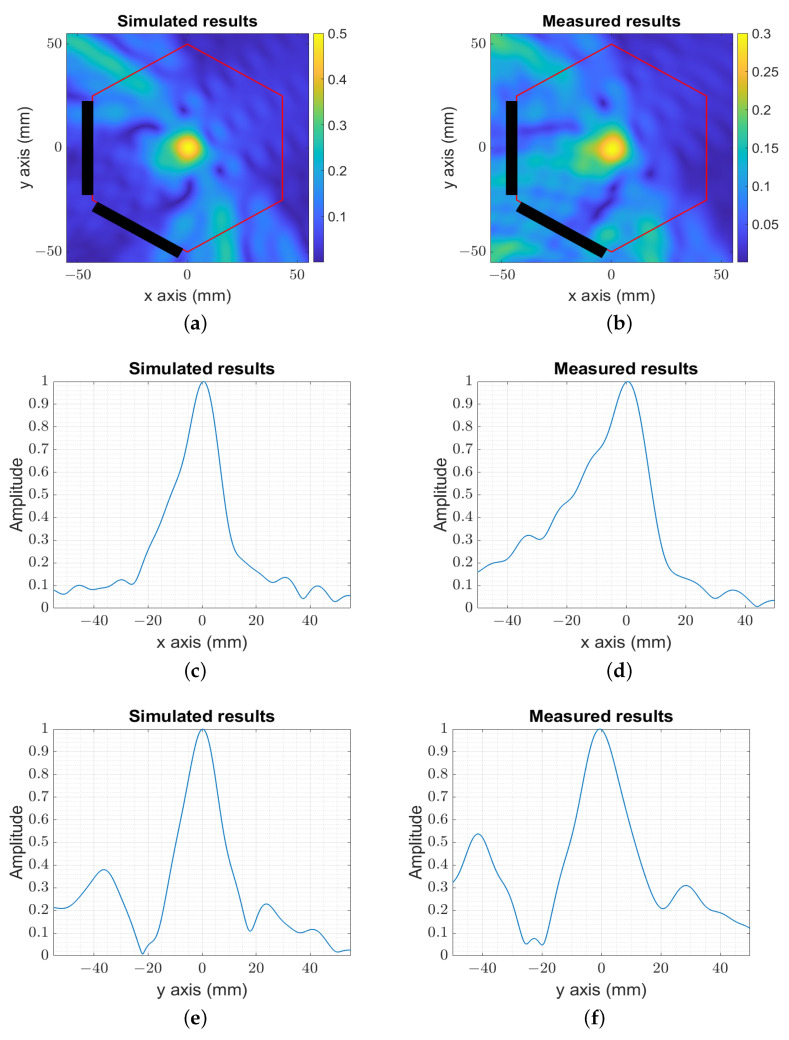
The image reconstruction of the simulated and measured target at the center. (**a**) The amplitude of the numerical centered image target. (**b**) The amplitude of the experimental centered image target. (**c**) The amplitude of the numerical image for y=0 mm. (**d**) The amplitude of the experimental image for y=0 mm. (**e**) The amplitude of the numerical image for x=0 mm. (**f**) The amplitude of the experimental image for x=0 mm.

The simulated and measured 2 × 2 signals were processed with (4) to locate the origin of the low-frequency (f≃1 kHz) signal generated with the photodiode. The numerical (Figure 5a) and experimental (Figure 5b) images for the target localized initially at the center of the hexagonal phantom were in agreement, and the low-frequency (f≃1 kHz) signal was properly located. The spatial resolution produced along the x-axis (Figure 5c,d) and y-axis (Figure 5e,f) was in the order of $l_{trn}^{elp}≃l_{lng}^{elp}≃10$ mm.

**Figure 6:**
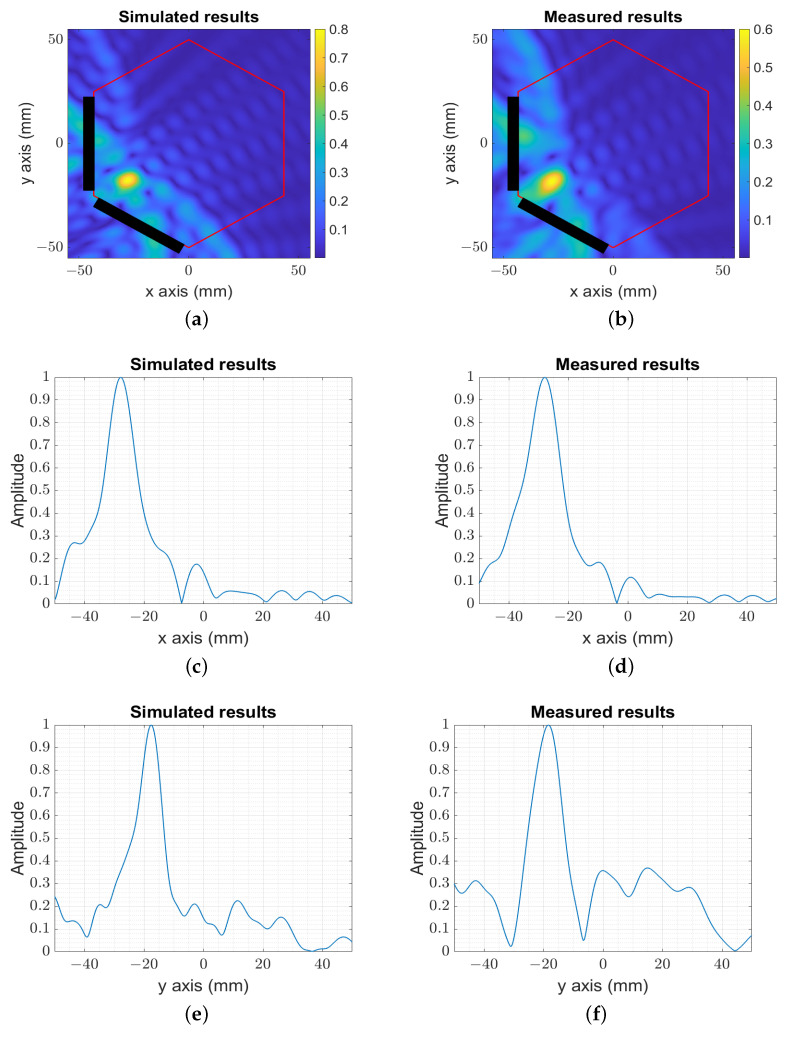
The image reconstruction of the simulated and measured target moved from the center. (**a**) The amplitude of the numerical image target moved from the center. (**b**) The amplitude of the experimental image target moved from the center. (**c**) The amplitude of the numerical image for y=−20 mm. (**d**) The amplitude of the experimental image for y=−20 mm. (**e**) The amplitude of the numerical image for x=−25 mm. (**f**) The amplitude of the experimental image for x=−25 mm.

For smaller distances from the probes to the target (numerical (Figure 6a) and experimental (Figure 6b)), the system showed a slightly better reconstructed resolution $l_{trn}^{elp}≃l_{lng}^{elp}≃7$ mm, as presented in Figure 6c,d for the x-axis and Figure 6e,f for the y-axis.

**Figure 7:**
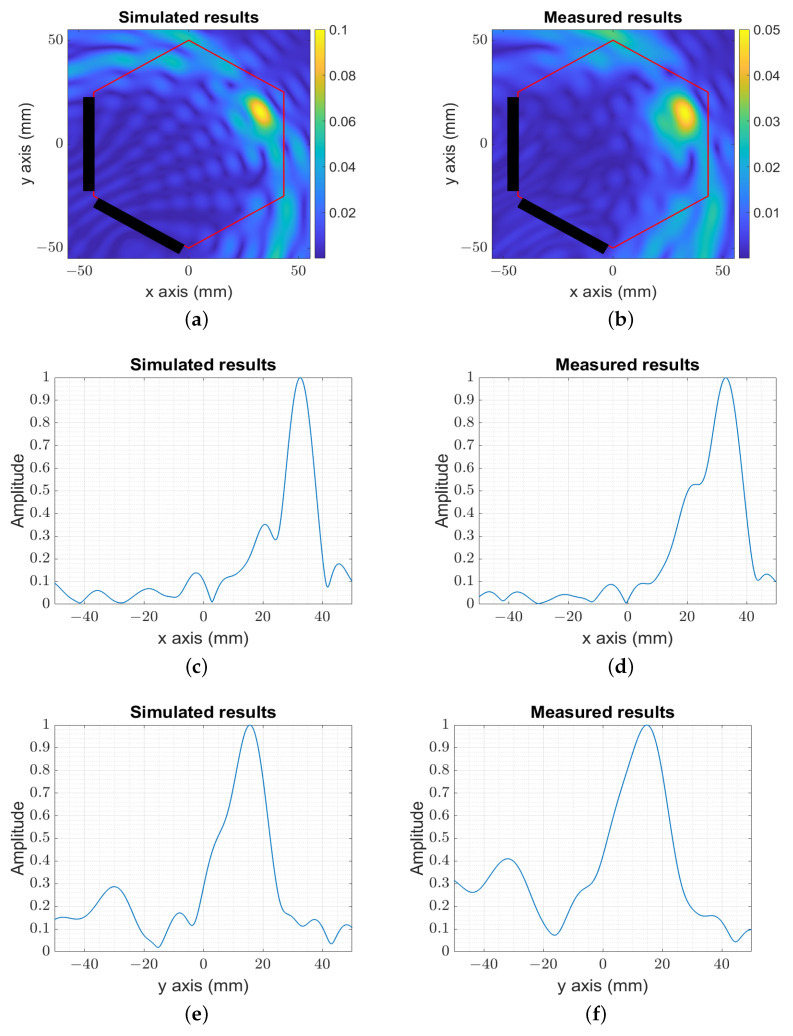
The image reconstruction of the simulated and measured target at the farthest position in the setup. (**a**) The amplitude of the numerical image target at the farthest position in the setup. (**b**) The amplitude of the experimental image target at the farthest position in the setup. (**c**) The amplitude of the numerical image for y=15 mm. (**d**) The amplitude of the experimental image for y=15 mm. (**e**) The amplitude of the numerical image for x=30 mm. (**f**) The amplitude of the experimental image for x=30 mm.

An additional location is studied in Figure 7, which corresponds to the farthest position within the phantom, to validate the reconstruction range in the reduced head model (diameter of 100 mm) compared to the realistic human head (diameter in the order of 200 mm). The reconstructed images (numerical (Figure 7a) and experimental (Figure 7b)) show the capability of locating the target at 100 mm from the probes with the reconstructed resolution, $l_{trn}^{elp}≃12$ mm and $l_{lng}^{elp}≃10$ mm, as presented in Figure 7c,d for the x-axis and Figure 7e,f for the y-axis.

In Figure 5, Figure 6 and Figure 7, from the transversal and longitudinal profiles of the reconstructed images, it was observed that there was still an approximate additional available dynamic range (between the peak of the profile and the noise level) of around 14 dB to 20 dB that should allow detecting the microorganism’s BMI.

The results confirmed the capability of the system to properly locate the actual origin of the signal, mimicking the functional bio-signal in the brain model in a variety of positions, from close to the probes to 100 mm away, which is equivalent to the radius of the major axis of the human head, with the spatial reconstruction resolution varying from 7 mm to 12 mm. Additionally, the system’s flexibility allows placing the probes as close as possible to the zone of interest (for example, in the case of Parkinson’s disease, close to the subthalamic nucleus (STN) and globus pallidus (GPi)).

## 4. Conclusions

This paper presented a novel methodology based on two UWB probes able to locate inside a region modeling a brain medium the modulated signal generated by a microtag approaching the functional activity produced by living microorganisms. The target detection was performed by the combination of the focusing capability of RF UWB probes with a low-frequency (f≃1 kHz) modulation technique, giving detection capabilities for objects of the order of the mm and positioning accuracies in the order of 10 mm. 

## Figures and Tables

**Table 1:** Comparison of analytical and experimental values of backscattered modulation index.

Backscattered Modulation Index @ 1GHz	Analytical Validation of the Microtag	Experimental Validation of the Microtag	Real Microorganism
${\tilde{\rho }}_{L_{i}}=\frac{Z_{i}^{PD}-{\left(Z_{a}^{SD}\right)}^{*}}{Z_{i}^{PD}\left(f\right)+Z_{a}^{SD}}$	$Z_{a}^{SD}=1-12j$ $Z_{ON}^{PD}=3\;\Omega$ $Z_{OFF}^{PD}=20,000\;\Omega$	Photodiode WL-SDCB SMT	Membrane 200μm×200μm
$BMI=\frac{{\tilde{\rho }}_{L_{ON}}}{{\tilde{\rho }}_{L_{OFF}}}$	≃0.045	≃0.040	≃0.005
